# Pituitary Extract as an Ecbolic

**Published:** 1913-01

**Authors:** F. F. Dollert

**Affiliations:** Milwaukee, Wis.; Associate Professor of Obstetrics in the Medical Department of Marquette University, Milwaukee; Associate Obstetrician in the Milwaukee Maternity Hospital; Member of the Obsterical Staff of Trinity Hospital, Milwaukee


### PITUITARY EXTRACT AS AN ECBOLIC.

By F. F. Dollert, Milwaukee, Wis.

*Associate Professor of Obstetrics in the Medical Department of Marquette University, Milwaukee; Associate Obstetrician in the Milwaukee Maternity Hospital; Member of the Obsterical Staff of Trinity Hospital, Milwaukee.*

There is probably no time in the routine experience of the general practitioner when greater anxiety is felt for the action of remedial agents than that period during labor, when the life of the mother and child is endangered from hemorrhage or delayed labor resulting from uterine inertia.

While the physicians have placed more or less confidence in the use of Ergot or large doses of quinine to carry them safely past the danger mark, neither agent can be relied upon in every case and even at best the results are frequently delayed to the point of greatest danger.

In casting about for an agent to fulfill this long felt want, I was attracted by the reports upon Pituitrin. According to the results of clinical and laboratory investigation, this preparation approaches the ideal as an ecbolic. After using Pituitrin in obstetrical practice, for both hospital and private work, covering a period of almost two years, I am fully convinced that it is the best and most reliable ecbolic known at the present time. Its action is superior to and of much longer duration than either ergot or quinine sulphate even if the latter is given in large doses, it frequently fails to produce any perceptible contraction.

I have used Pituitrin not only to induce labor, but during the natural progress to increase the power of contraction. Have also given it after the termination of labor in cases of hemorrhage or where there is a tendency or possibility of hemorrhage. To induce labor 1 to 2 cc. (16 to 32 minims) is given subcutaneously, which is usually sufficient to produce the desired effect. During labor, if the pains become weak and irregular, 1 cc. (16 minims) given hypodermically usually stimulate powerful contraction, which is apparently normal in every respect.

After the first stage of labor, if the uterus is soft and there is considerable oozing of blood, an injection of 1 cc. of Pituitrin will cause prompt contraction of the uterus and cessation of bleeding. In casts of post-partum hemorrhage its use as above stated will keep the uterus contracted, thereby controlling hemorrhage and have a general stimulating effect upon the patient.

Its superiority over Adrenalin as a stimulant has caused me to abandon the use of the latter to a very great extent and so far as I have been able to determine there is absolutely no harm resulting from its use either to the mother or child.

The following reports of cases represent the many in which I have observed brilliant results with Extract of Pituitary Body:

Case 1.—Mrs. S. Age 31. Primipara. Small and delicate. In labor twelve hours. Examination: Child in R. O. P. position, head engaged, cervix dilated. Liq. Amini and Meconium stained, fetal heart weak and irregular, applied forceps and delivered, head remaining in posterior position. After resuscitating the child, attention was directed to the patient who was bleeding profusely, fundus uteri soft and relaxed and puse 120 threading. The patient was given 1 cc. Pituitrin subcutaneously and within a few seconds the uterus began to contract and all bleeding ceased, pulse became slow and full and the patient soon stated that she felt better.

In this case, as well as many others, the Pituitrin stopped the hemorrhage and produced a general stimulating effect as evidenced by the quality of the pulse and the general appearance of the patient.

Case 2.—Mrs. K. Age 27. Normal labor. Examination revealed normal progress of labor, although very slow, and pains at irregular intervals. At 8:00 P. M. gave 1 cc. Pituitrin, hypodermatically. Within fifteen minutes firm contractions began with regularity and within six hours the patient was delivered of a healthy eight and a half pound child. The fundus remained firm throughout the third stage. The patient made a rapid recovery and there was no evident effects from the medicine upon the child.

Case 3.—Mrs. K. Age 29. Patient three and one-half months pregnant, suddenly developed pains in the back, slight hemorrhage from vagina; vaginal examination revealed slight dilatation with some projection at point of an old laceration of cervix cervix, which had been repaired but evidently gave way at the lower end of the rupture. It is very evident that miscarriage was inevitable. The fetus was promptly delivered but the placenta failed to come away even with slight traction of the cord. About midnight the patient received 1 cc. Pituitrin hypodermically, contraction were prompted and continued for several hours, although they were not vigorous. At 10:30 next morning the placenta was part way out of the lacerated portion of the cervix and patient received another injection of 1 cc. Pituitrin which was followed by considerable contraction. At 4:00 P. M. the patient received the third dose 1 cc. Pituitrin and another at 9:00 P. M. Following the latter dose vigorous contractions were observed and within a short time the entire mass was delivered intact.

In this case the treatment seemed to be ideal. By being able to control the contractions by the size and interval of dose, one may produce a mild continuous contraction, which is a much safer plan than to undertake to make immediate delivery by manual manipulation or instrument. Two days later the cervix was found to be closed and firm, the fundus contracted, flow normal, no temperature nor pulse.

Case 4.—Mrs. S. Age 19. Patient very delicate, bony structure small. After eleven hours of apparent normal labor, the pains became weak, irregular and intermittent. Gave 1 cc.

Pituitrin and in about five minutes the patient noticed the pains returned. The contractions soon became vigorous and regular and in five hours from the time of injection patient gave birth to a normal child. Immediately after delivery the uterus became soft and contraction weak. Placenta was expelled in forty-five minutes. Uterus remained soft and there was considerable hemorrhage. Gave 4 cc. Fluid Extract of Ergot, which did not seem to influence the condition; gave 1 cc. Pituitrin hypodermatically and within forty-five seconds contractions were resumed and the organ remained firm and the patient made uneventful recovery.

Case 5.—Mrs. V. Age 24. Patient poorly developed and anemic with history of painful menstruation and profuse hemorrhage lasting three or four days, after which the patient suffered from weakness, backache and headache. Examination revealed a large soft retroverted uterus. Patient objected to operative interference, and was told to return at menstruation period, when 1 cc. Pituitrin was injected subcutaneously. She returned the next day and received another injection of 1 cc. Pituitrin. On the third day the flow was so scant that no more injections were given. As the patient did not suffer from the usual flow, she felt much stronger and was relieved of practically all of the painful symptoms which she had experienced at former periods, which clearly demonstrates the marked effect of this treatment.

---

### CLINICAL SOCIETY OF OF NEW YORK POLYCLINIC MEDICAL SCHOOL AND HOSPITAL

*Dr. A. T. Morrow Presented a Case of Ruptured Bladder*

The patient, a male 22 years old, had pelvis and abdomen crushed between two freight cars two weeks ago. Brougth to the hospital in shock. Only signs of injury externally were large fluctuating mass over right side above thigh, and a slight swelling over the pubis. Pure blood was obtained by catheter.

Operation revealed all tissues contused right rectus completely torn across and free urine in peritoneal cavity, and no tear in bladder. The latter was incised and finger found intraperitoneal tear in upper left quadrant. Also partial rupture of sigmoid to mucus membrane. Cecum was torn from its attachment for three inches and posterior peritoneal lining ripped from side to side. Torn parts were sewed and catheter introduced. At present pulse and temperature are normal. Ecchymoses prominent over abdomen. Catheter is still in place and right rectus is separated awaiting a larger operation.

DR. JOHN A. BODIE, REMOVAL OF CANCER OF TONSIL THROUGH INCISION IN NECK

The growth began as a small erosion on the tonsil, and was treated medically four weeks. After having spread over the whole tonsil pillars of fauces, the folds and tongue, great advance was shown in this short time. The patient came for radical operation two weeks ago. The method used was that from without, transforming a cavity operation into a surface one, and enabled a thorough cleaning out. A ligature was thrown about the external carotid, and all the glands of the neck including the submaxillary gland which is a double gland with lymphatics on the inside of it were removed. The lower jaw was sawed through obliquely so that the opposing forces of the masseter and external pterygoid would subsequently hold the edges in apposition. In this particular instance, extra strength was afforded by the insertion of a Lane plate. The two tonsils, part of the floor of the mouth, pillars of the fauces and tongue were removed. The patient is quite comfortable now; a few days after the operation, but the prognosis for life is bad. The richer the supply of lymphatics in a region, the more rapid the growth. The question of removal of a part of a growth for microscopical examination came up and both Dr. Bodine and Dr. Bainbridge condemned it. In every case a large opening should be made and complete enucleation done—not piece meal work. Dr. Bodine reported a case of a man with a very small ulcer on the tip of his tongue, from which a large swelling appeared on the side of the neck. These growths spread like wild fire when they are tampered with. Dr. Bodine said that the average time of relief for peripheral operations is ten months, and wished to go on record as saying that 70% of patients have cancer anywhere in the body die a cancerous death.

DR. JOHN A. BODINE.—AVULSION OF PROSTERIOR ROOT FOR TRIFACIAL NEURALGIA

The patient, a woman of 63, sought relief for Tic Douloureux by all the ordinary measures and by alcoholic injections without result. Permanent operative cure depends upon the inability of the nerve to regenerate which occurs when the nerve has no neurilemma. The sensory root of the fifth nerve beyond the Gasserian ganglion has no sheath and therefore if it is cut in that position it cannot regenerate. The death rate from this operation is 3% to 4%. In the operator's opinion it is not very difficult to perform. A prophylactic ligature is placed around the external carotid artery, an incision recommended by Cushing made over the zygoma, and the hemmorhage from the middle meningeal artery controlled by plugging it down in its bony canal. The white glistening root of the fifth nerve beyond the Gasserian ganglion is easily seen and severed. The Anaesthetic can now be discontinued since there is no sensation for the rest of the operation. This patient on the following day, had a facial parylisis which soon cleared up, and since the nerve at operation was not cut the paralysis was probably due to traction during operation. The motor root that lies immediately above the Gasserian ganglion must be avoided, otherwise paralysis of the muscles of mastication ensues. The patient is now free from pain, with slight hyperesthesia. The day of Abbe's operation is gone—there is only a small field for peripheral operations. Alcohol injections give just as much relief as any peripheral operation. Dr. Kennedy congratulated Dr. Bodine on his able defense of the operation for the resection of the sensory root of the fifth nerve. The speaker had considerable experience with this operation and spoke highly of it. He opposed alcohol injections on the ground of complications. Dr. Keller disproved this by quoting favorable statistics in a large number of cases. The time of relief in skilled hands was from six months to two and half years, which is a very satisfactory result and a better showing than in peripheral operations.

Dr. John A. Bodine.—Cystic Tumor of Cerebellum

Brain tumor operations are increasing every year, but operations for removal of a cyst, is sufficiently rare to justify showing this case. Four months prior to the operation, which took place two years ago, this middle aged man suffered loss of health from no assignable cause. Later became more irritable, eyesight weakened, and one day while walking fell unconscious in the street. The symptoms of increased intracranial pressure now developed. Vomiting, headache, progressive blindness, loss of equilibrium, with falling to right side, paralysis of muscles of extremities, disturbances of special senses and reflexes, incontinuance of urine and faeces, and extreme pain. A spinal puncture, was suggested of which Dr. Bodine, emphatically disapproved. Since the fluid in this case is of no diagnostic value, and the cerebellum is already pushed down into the foramen by the existing increased pressure the withdrawal of fluid may impact the brain, still further in the opening and instant death follow. The patient was operated upon in a sitting posture and a cyst the size of a thumb was found which burst. Its lining being too thin to be cut away, surrounding portions of the cerebellum were removed. The patient made a rapid recovery and is in good health. Dr. Bainbridge raised an interesting question of the effect of tumors upon the moral of the individual. He thought that possibly care should be exercised in convicting these cases of cerebral tumor for acts committed while suffering from their affliction. Dr. Kennedy suggested that the field of operation in brain work could be kept in better condition while operating if the surgeon used the suction apparatus as used by dentists.

---

Philadelphia, Dec. 27, 1912.

EDITOR JOURNAL-RECORD OF MEDICINE:

The following communication has been sent to the editors of "The Virginia Semi-Monthly Medical Journal; The Southern Medical Journal and the Charlotte Medical Journal:"

The following extract from an article: "The Negro and His Health Problems," by Dr. J. Madison Taylor, appearing in the Medical Record of September 21st, 1912, has excited a degree of unfavorable criticism from different sources, which contend that the imputation is not fair to the negro race.

An important observation made by several Southern physicians is to the effect that sexual lawlessness, remsulting in lynchings, is to be explained thus: The male negro is nearly always infected with gonorrhoea. He neglects treatment habitually. Chronic prostatic irritation ensues. Thus sexual impulses frequently become overmastering. Alcohol particularly inflames this state and likewise dethrones reason; hence the first female he meets he is tempted to assault. This is often a white female; the fury of vengeance of white men is excited and disastrous results follow the negro."

The idea of the comparative prevalence of gonorrhoea in the race is a generally accepted statement among medical men of the North, but I have been unable to verify it by any statistics or other authorative sources. May I ask you to bring the matter editorially before your subscribers asking for contributions on the subject. An answer to the following questions may be of assistance in getting at the pith of the matter.

In your personal medical experience as compared with the white race:

1. Is gonorrhoeal urethritis more prevalent among the adult negro male population?

2. Are its sequeals, pyosalpynx, pelvic abscesses, etc., more common among the adult negro population?

3. Is the proportion of gonorrhoeal ophthalmia greater among negro newborn?

4. Is the disease more prevalent among the city or country negro population?

5. Is the disease as virulent in the colored race?

6. In chronic gonorrhoea and its sequeale, gleet, prostatitis and seminal vesiculitis more prevalent in the negro male?

7. Is this condition responsible or the sexual lawlessness mentioned in the previous quotation from the article of Dr. Taylor?

Realizing that you will appreciate that fairness and not prejudice is instrumental in instigating the above inquiry; I hope through your journal will aid us in answering the question?

Dr. Searle Harris has suggested that I write to you personally as one in a position to give the desired information, and any assistance will be greatly appreciated.

Very truly yours,

WM. A. STEELE,
Consulting Urologist, Samaritan Hospital
3300 North Broad Street.

